# Non-operating Room Anesthesia Services From a Tertiary Specialized Center in Saudi Arabia: An Overview With Literature Review

**DOI:** 10.7759/cureus.82404

**Published:** 2025-04-16

**Authors:** Rayan Muawad, Abdullah A AlDhuwaihy, Albraa K Aldawood, Juwan Al Musma, Marwan A Almalki, Mohammad Alrashed, Abdullah A Al Harbi

**Affiliations:** 1 Department of Pediatric Anesthesia, King Abdullah Specialist Children's Hospital, Ministry of National Guard Health Affairs (MNGHA), Riyadh, SAU; 2 College of Medicine, King Saud University, Riyadh, SAU; 3 College of Medicine, King Saud Bin Abdulaziz University for Health Sciences, Riyadh, SAU

**Keywords:** adverse events, anesthetic services, healthcare systems, non-operating room anesthesia, patient safety, scheduling efficiency

## Abstract

Introduction

Non-operating room anesthesia (NORA) is rapidly expanding as it provides a less invasive option to the conventional operating room (OR), yet increases the efficiency and reduces the burden of OR resources. It is frequently used in well-equipped rooms in areas such as cardiology, radiology, and endoscopy, allowing patients to be treated efficiently. Nonetheless, with the complexity of cases and the challenges of the non-OR environment, NORA cases have higher risks of morbidity and mortality, highlighting the need for standardized protocols, special training, and safety measures to mitigate such risks.

Methodology

The data were obtained from the IT department at King Abdullah Specialist Children Hospital, Ministry of National Guard Health Affairs (MNGHA), on NORA cases from 2023 and 2024. Databases such as PubMed, Scopus, Web of Science, and Google Scholar were used to conduct the literature review. The inclusion criteria involved non-OR procedures in a tertiary hospital and studies published from 2000 to 2025. OR-based anesthesia, studies conducted outside of the timeframe, and non-indexed and non-English research were excluded.

Data and descriptive analysis

By assigning specified time periods for procedures, we developed an organizational strategy-based NORA system that enhanced scheduling and service delivery while lowering cancellations. This strategy ensures improved resource use, increases efficiency, and prevents emergency services from being misused. Although NORA cases have increased overall, indicating rising demands, the variation in the service trends points to the need for additional research to maximize resource allocation and meet changing patient needs.

Conclusion

NORA is increasingly becoming an essential asset within healthcare services, facilitating improved efficiency and better patient outcomes. However, to achieve more resourceful utilization of NORA, improvements in scheduling and service delivery must be implemented at an institutional systemic level. Our study demonstrated that implementing an efficient scheduling system in NORA services can lead to significant improvements in healthcare provision. However, many challenges still impede the effective implementation of NORA, and a continued review of data and patient feedback is essential.

## Introduction

Over the past decades, physicians have been able to treat patients through less invasive methods outside of the operating room. For instance, non-operating room anesthesia (NORA) involves administering anesthesia outside the OR for procedures such as those in interventional cardiology, endoscopy units, or radiology departments. Unlike operating room anesthesia, NORA is done in busy, fast-moving settings by just a single anesthesia provider [1]. NORA procedure rooms are prepared and equipped for specific procedures, personnel, and locations, ensuring that anesthesia delivery in these settings is the same high standard as those in the operating room [2]. As the number of cases grows, along with existing system challenges, the risk of adverse events increases. NORA cases have increased incredibly and are expected to reach 50% or more of all anesthesia cases within the next decade [1]. NORA patients are generally older and more medically complicated than those in traditional operating rooms.

Research indicates that NORA cases have higher rates of morbidity and mortality, with a greater incidence of preventable adverse events compared to OR cases. This increased risk is often linked to inadequate management of these adverse events, as staff in non-OR settings are generally less prepared to manage anesthesia-related complications than OR staff [3]. It holds significant importance in Saudi Arabia as it aims to establish a higher quality of medical care by contributing to reducing strain on operating room resources, establishing efficient medical procedures, and meeting the needs of an increasing and diverse number of patients. NORA has been shown to expand the scope of anesthesiology practice to manage more complex patients and procedures outside the operating room and minimize the risk to patients by reducing serious complications and improving patient-specialist satisfaction. Additionally, it can offer both time and cost savings compared to traditional operating room procedures. As it takes place outside the OR, it saves resources such as operating room staff and equipment. With fewer staff members involved and a higher rate of patient turnover, NORA facilitates greater resource efficiency and healthcare productivity [4]. In contrast, NORA showed shorter length of stay and lower mortality in specific conditions, particularly in association with minimally invasive cardiac procedures performed in the catheterization lab [5]. Since NORA has unique challenges and risks compared to operating room anesthesia, it requires standardized guidelines and protocols to ensure patient safety, because the environment and personnel may be less familiar with anesthesia [4]. Additionally, it might raise costs for the institution if anesthesia services are not managed properly through appropriate scheduling and efficient use of staff and facilities [6]. There is a significant need for specialized training to prevent the serious safety risks associated with NORA, such as airway compromise, aspiration pneumonia, and dental injuries [7]. Without proper expertise and preparation, these potentially life-threatening complications can undermine patient safety. Additionally, anesthesiologists themselves are at risk of exposure to high levels of radiation, which should be decreased through the use of effective personal protective shields [3]. We conducted this study because it provides new insights into the field of NORA, offering a fresh perspective on an area that has not been thoroughly explored in Saudi Arabia. This research aims to optimize services and facilitate improved patient outcomes.

## Materials and methods

This study was implemented at a tertiary specialized center during the period (2023-2024). The study utilized a retrospective data collection approach, wherein data were sourced from the Information Technology (IT) Department at King Abdullah Specialist Children’s Hospital, under the umbrella of the Ministry of National Guard Health Affairs (MNGHA), located in Riyadh, Saudi Arabia. The data encompassed all NORA procedures performed during the calendar years 2023 and 2024 and are summarized in Table 1. The primary objective of data collection was to analyze trends, patterns, and clinical practices associated with anesthesia services delivered outside the traditional operating room environment.

The dataset specifically included procedures from a wide array of clinical service categories where NORA is commonly administered. These included gastrointestinal (GI) procedures, bronchoscopy, angiographic interventions (Angio), endoscopic procedures, oncology-related diagnostic or therapeutic procedures, radiation therapy sessions, nuclear medicine (NM) investigations, computed tomography (CT) imaging, and magnetic resonance imaging (MRI). Each of these service categories represents a clinical area where anesthesia support is often required to ensure patient immobility, procedural success, and safety.

Data variables extracted included the type of procedure, patient demographics (such as age and gender), anesthesia technique used, duration of anesthesia, location of procedure, and any reported perioperative complications. This detailed breakdown allowed for a comprehensive evaluation of the scope and complexity of NORA services provided at the institution.

To supplement the empirical data analysis, a comprehensive literature review was conducted to contextualize findings within the broader academic and clinical landscape. A systematic search strategy was developed to ensure a robust and inclusive review of existing scientific evidence related to NORA practices. The literature search was conducted using four major scientific databases: PubMed, Scopus, Web of Science, and Google Scholar. These databases were chosen due to their extensive coverage of biomedical, clinical, and multidisciplinary research. The search strategy incorporated a combination of relevant keywords, Boolean operators, and Medical Subject Headings (MeSH) terms, including but not limited to “non-operating room anesthesia,” “pediatric anesthesia,” “out-of-operating room procedures,” “imaging anesthesia,” “endoscopic anesthesia,” and “NORA safety outcomes.”

Inclusion and exclusion criteria

To maintain the scientific rigor and relevance of the literature review, a clearly defined set of inclusion and exclusion criteria was established prior to the screening of articles.

The inclusion criteria encompassed original research articles, reviews, meta-analyses, or clinical practice guidelines that specifically addressed NORA procedures, publications focusing on anesthesia administration during procedures in service areas such as GI, bronchoscopy, angio, endoscopy, oncology, radiation, nuclear medicine, CT, and MRI, and articles published in the English language between January 2000 and January 2025 to ensure both historical depth and contemporary relevance.

The exclusion criteria were as follows: studies focusing on anesthesia delivered within operating room environments, as the scope of this study is strictly limited to non-operating room settings; articles falling outside the designated publication timeframe (i.e., prior to January 2000 or after January 2025); studies that were not indexed in the selected electronic databases, to ensure the credibility and academic reliability of the sources; articles published in languages other than English, due to translation limitations and the need for standardized review; and publications that did not present sufficient methodological clarity, statistical rigor, or clinical applicability to the objectives of this study.

Study strengths

This study offers valuable institutional insight into the implementation of a structured scheduling system for NORA, highlighting its role in improving procedural coordination, patient throughput, and resource utilization. By analyzing departmental volume trends over time, the study captures evolving procedural demands and service distribution across specialties. These findings not only reflect local operational success but also align with broader global trends, emphasizing the growing importance of NORA in contemporary healthcare. Furthermore, the scheduling framework presented is scalable and adaptable, offering a practical model that other institutions can adopt to optimize their own NORA services. The study also emphasizes patient safety through improved workflow efficiency, reflecting current best practices in anesthetic care outside the traditional operating room.

Study limitations

Despite its contributions, the study is limited by its retrospective and descriptive design, which does not include inferential statistical analysis or hypothesis testing. As a result, causal relationships cannot be confidently established. Although the intervention, a structured scheduling model, is described, the study does not include a quantifiable pre- and post-implementation analysis of key outcome metrics, such as complication rates or patient wait times. Additionally, since the data were collected from a single tertiary institution, the generalizability of the findings to other healthcare systems with differing resource constraints is limited. Another important limitation is the initial lack of strong integration between the literature review and the discussion of findings, which may impact the perceived academic originality of the work. Finally, the study does not incorporate qualitative perspectives from healthcare providers or patients, which could have added depth to the understanding of the intervention’s broader impact on care delivery and experience.

Through this structured and methodical approach, the research aims to generate a detailed and evidence-informed understanding of NORA practices, contributing valuable insights for clinical optimization, resource planning, and patient safety enhancement in anesthesia services.

## Results

Data and descriptive analysis

As part of this quality improvement initiative, a structured NORA system was developed and implemented, grounded in a well-defined organizational strategy, as outlined in Table 1. This system was designed to enhance procedural coordination, improve scheduling efficiency, and optimize the utilization of hospital resources. The strategy involved the allocation of specific time slots and designated periods throughout the week for each service category that requires NORA support. The system ensures a standardized scheduling mechanism where procedures are systematically aligned with departmental availability, allowing for seamless integration across clinical units.

**Table 1:** Structured scheduling framework for NORA services NORA: Non-operating room anesthesia

Time	Sunday	Monday	Tuesday	Wednesday	Thursday
8:00	Radiation	Radiation	Endoscopy	Radiation	Radiation
9:00	Radiation	Radiation	Endoscopy	Radiation	Radiation
10:00	Radiation	Radiation	Endoscopy	Radiation	Radiation
11:00	Oncology	Oncology	Endoscopy	Oncology	Oncology
12:00	Oncology	Oncology	Medical Imaging-Angio	Oncology	Oncology
13:00	Endoscopy	Endoscopy	Medical Imaging-Angio	Endoscopy	Endoscopy
14:00	Endoscopy	Endoscopy	Medical Imaging-Angio	Endoscopy	Endoscopy
15:00	Endoscopy	Endoscopy	Medical Imaging-Angio	Endoscopy	Endoscopy
16:00	Urgent Patients	Medical Imaging	Urgent Patients	Medical Imaging	Medical Imaging

This methodical approach has led to tangible improvements in service delivery and patient management. Key benefits observed include a reduction in case cancellations, expedited patient bookings, shorter turnaround times for results, and a decreased burden on emergency services. A major contributing factor to this improvement is the clear demarcation between elective and emergency cases, which prevents non-urgent procedures from being inappropriately classified as emergencies, a common issue in the absence of a structured system. This misclassification previously contributed to overloading the emergency team, missed appointments, and inefficient resource use. The new system addresses these challenges by ensuring each case is scheduled appropriately, improving workflow, increasing patient satisfaction, and enhancing overall service quality.

Moreover, the system supports proactive planning and resource alignment, minimizing scheduling conflicts and overbooking scenarios. It provides a reliable framework that facilitates smooth coordination between anesthesia teams and various procedural departments, ensuring timely service delivery and optimal use of institutional capacity.

The effectiveness of this approach is reflected in the quantitative analysis of NORA service volumes between 2023 and 2024, as shown in Table 2. GI procedures showed a 5.6% decline, decreasing from 467 cases in 2023 to 441 in 2024. This reduction, while moderate, may suggest either a shift in procedural demand, a change in referral patterns, or increased reliance on alternative diagnostic modalities. Services that utilized radiation as a mode of therapy or diagnostics noticed a decline in cases between 2023 and 2024. CT and MRI services saw a modest 4.4% decrease and a 5.6% decrease, respectively, in cases. This slight drop could indicate a redistribution of imaging preferences toward MRI and CT services or a shift in patient demographics and diagnostic criteria. Furthermore, radiation therapy noticed a significant diminution in cases, with an approximate 46% decline in cases from 936 to 427 in 2024. Moreover, this decrease could be multifactorial, potentially influenced by equipment downtime, evolving clinical guidelines, or a more selective approach to MRI utilization in the face of increasing demand in other imaging modalities. However, an increase in cases was also noted in multiple departments, which may reflect multiple improved capacities, increased demand with improved systemic structure, and better use of previously underutilized anesthesia-supported services.

**Table 2:** Anesthetic services in non-operating rooms (2023-2024)

Year	NORA									
	Angio	Endoscopy		Oncology	Radiation	NM	CT	MRI	Total	
		GI	Bronchoscopy							
										2023	834	467	53	971	936	67	206	2149	5683		
										2024	962	441	55	990	427	120	197	2029	5221		
Total	1796	908	108	1961	1363	187	403	4178	10904		

Angiography demonstrated a substantial 15.4% increase, growing from 834 to 962 cases. This upward trend could reflect enhanced capacity within the imaging department, broader clinical indications, or improved patient throughput due to the structured scheduling system. Bronchoscopy remained relatively stable, increasing marginally by 3.8% (from 53 to 55 cases). The minimal change may indicate consistent demand and capacity, suggesting that this service is operating near its equilibrium point. Similarly, oncology-related procedures rose from 971 to 990 cases, an increment of approximately 2%, possibly indicating a slight increase in patient caseload or procedural complexity without a substantial change in departmental volume. On the other hand, the Nuclear Medicine Department exhibited the most pronounced growth, with a dramatic 79% growth in cases in 2024. This dramatic rise likely reflects major institutional or technological advancements, such as the introduction of new treatment protocols, increased patient referrals, or improved scheduling flexibility under the new system. This growth may also correspond to expanding clinical applications or better integration of anesthesia support into previously underutilized services.

The aggregate volume of NORA cases decreased from 5,683 in 2023 to 5,221 in 2024, representing a 4% overall decrease in service demand. This trend is graphically represented and can be attributed to effective case stratification, which has contributed to improved procedural organization. By assigning each procedure to designated time slots based on clinical priority and departmental availability, the system has minimized inappropriate or biased utilization of NORA services, ensuring more efficient and equitable use of anesthesia resources.

## Discussion

This study was informed by a structured scoping review based on the Arksey and O’Malley framework. We searched PubMed, Scopus, and Google Scholar using a broad set of keywords related to NORA. Of the 132 identified articles, 44 were included after screening based on relevance to procedural categories, safety concerns, and healthcare system integration.

NORA represents a cutting-edge sub-specialty within anesthesiology, demanding further comprehensive research to elucidate its intricacies. This need is particularly pronounced in Saudi Arabia, where studies are required to delve into procedure outcomes, associated complications, and safety measures. NORA refers to anesthesia administered outside the traditional OR [1], often in remote locations within the hospital. It demands a high level of expertise and skill [8]. The settings of NORA encompass a variety of procedures across multiple specialties. These include gastroenterology procedures, psychiatric treatments, neurosurgical procedures, dental procedures, radiology for both diagnostic and interventional purposes, and pulmonary procedures, such as bronchoscopy, oncology, and cardiology interventions [9]. NORA procedure rooms are specifically constructed and equipped to accommodate unique medical interventions, and they are tailored to the needs of the procedures, the expertise of the staff, and the specialized location required for these interventions [2].

Studies examining the prevalence of NORA have noted that it currently constitutes one-third of all anesthesia cases [10]. Projections suggest that it could represent half or more of anesthesia cases within the next decade [11]. The significance of NORA lies in its ability to reduce the need for surgical procedures, particularly for patients who require non-surgical interventions. One study concluded that NORA cases have lower mortality rates, approximately 0.02%, compared to OR anesthesia (ORA) cases, which have a mortality rate of about 0.04% [10].

A large number of NORA procedures are considered minimally invasive day-care procedures [12]. The most common NORA procedures include endoscopies, with esophagogastroduodenoscopy (EGD) and endoscopic retrograde cholangiopancreatography (ERCP) being the most prevalent [13]. The agents used in these procedures vary, including lidocaine, benzocaine, and propofol [14]. Additionally, NORA procedures in bronchoscopy within interventional pulmonology require adequate suppression of airway reflexes, which can be achieved with a 2% lidocaine solution mixed with epinephrine-soaked [2]. Similarly, in interventional cardiology, short-acting inhalational, intravenous, and local anesthesia can be employed [2]. Anesthetic methods in interventional radiology include epidural, general, local anesthesia, and opioids, as well as anxiolytics [15]. One technique for light anesthesia involves peripheral nerve blocks; for example, the phrenic nerve block is used for CT-guided lung biopsies [16]. In MRI suites, minimal sedation is often provided, and light sedation can be achieved with incremental doses of midazolam or fentanyl, and hypnotics and analgesics such as remifentanil or other opioids can be combined [2].

In pediatric patients, one report identified the overall rate of adverse events for pediatric anesthesia cases outside the OR as 6.4%, encompassing 37 institutions and 49,836 cases [17]. Mohammed et al. demonstrated that prolonging perioperative pain control during pediatric cardiac catheterization is achieved with deep sedation alongside caudal analgesia [18]. For lumbar puncture procedures, which are frequently performed in pediatric patients, local anesthesia such as EMLA cream and 1% lidocaine injections without adrenaline are commonly used [2]. Similarly, the standard of care for bone marrow aspiration procedures includes the application of local anesthesia [2]. In obstetrics and gynecology, oocyte retrieval can be performed under paracervical, spinal, epidural, intravenous sedation, and general anesthesia [19]. A randomized double-blind placebo-controlled study investigating the efficacy of paracervical blocks in oocyte retrieval found that pain scores were reduced by 38.9% and 51.4% compared to placebo and no local injection, respectively [20].

There are several social and organizational challenges in implementing NORA procedures. Social challenges include poor communication [21,22], while organizational challenges encompass a shortage of essential equipment [22-26], insufficient drug reserves [3,10,21], and non-standardized workstation setups [27]. Inadequate lighting, lack of electrical outlets [24], loud noise levels [28-30], and cramped workspaces [9,25,31,32] are notable environmental concerns in NORA settings. Additionally, outdated equipment and limited postoperative monitoring capabilities are significant technological issues [3,21,23,29,33,34]. A survey on monitoring techniques during sedations by non-anesthesia providers highlighted inconsistent application of basic monitoring principles [35]. It is essential that intraoperative monitoring in NORA environments adheres to the same standards and qualities as those in the OR [30].

A study showing severe adverse events in NORA compared to the OR found that 50% of adverse events were classified as severe in the OR, while 58% were in NORA settings [36]. Additionally, studies have shown that anesthesiologists' exposure to radiation can exceed that of interventional specialists [37,38]. Research indicated that the commonest minor adverse outcome in NORA cases is postoperative nausea and vomiting, along with inadequate pain control, and the major adverse outcome is serious hemodynamic instability. An analysis of subcategories within NORA revealed that cardiology and radiology procedures had an increased mortality rate of 0.05% [10]. Another observation was that substandard care was more prominent in NORA, with many complications potentially preventable through better monitoring [12]. Cravero et al. reported on the occurrence and nature of adverse events in NORA for over 30,000 pediatric patients, with no deaths recorded. The most prominent type of complication was respiratory-related. For every 89 NORA procedures, there was an event that could have been harmful [39]. Working under such conditions may contribute to increased stress and fatigue among anesthesiologists [40].

The adoption of a structured NORA system has significantly enhanced healthcare delivery within our institution, aligning with global trends that underscore NORA’s growing importance in modern clinical practice. Multiple studies have projected that NORA will account for approximately 50% of all anesthesia cases in the coming decade [1,11], a trend reflected in our data showing an approximate 4-5% decrease in total NORA cases from 2023 to 2024. Although a decrease in total NORA cases was recorded, multiple departments have noticed an increase in NORA cases. Along with the global projection of an increase in NORA demand, this growing need supports prior literature, suggesting NORA’s fundamental role in addressing procedural needs outside the traditional OR [10,12]. Our findings support these reports, primarily with notable increases in angiography (15.4%) and nuclear medicine (79%) cases - matching global innovation and expanding indications for NORA in these specialties [15,16]. The literature has emphasized the need for high-quality anesthetic care in such settings due to the sophistication of procedures and vulnerable patient populations [2,17], reinforcing the importance of our structured scheduling strategy in reducing safety risks.

Furthermore, the decline observed in MRI, CT, and radiation therapy cases reflects changing institutional priorities and diagnostic shifts, as also observed in other studies that report changing imaging preferences and resource redistribution [22,23]. This distinction emphasizes the need for dynamic resource planning and adaptive scheduling models - concepts endorsed in recent operational efficiency studies [41]. Our organizational scheduling framework has shown substantial improvements, including reduced cancellations and improved workflow efficiency - outcomes reflected in prior research that associates systematic scheduling with improved patient throughput and reduced overrun times [41-43]. For instance, the increased throughput in angiographic procedures (15.4%) aligns with literature highlighting the value of predefined procedural time blocks in minimizing delays and improving coordination [6,10].

The variability in growth rates across services in our dataset further supports literature citing the heterogeneous nature of NORA implementation and the need for modified strategies per specialty [9,30]. Radiation therapy’s abrupt decline may reflect the requirement for better service integration and staff coordination - a critical factor in safety and efficiency highlighted in studies addressing technological and organizational barriers in NORA environments [21,27].

Our results also support previously identified concerns about adverse event risks in NORA environments [3,10,36]. Although preoperative complication data were not deeply analyzed in this study, the expansion of service volumes necessitates enhanced monitoring standards, cross-training, and radiation safety measures, as reflected by numerous authors [3,7,38]. The increase in more complex procedures, such as those in oncology and interventional cardiology, mandates attention to adverse event prevention and patient safety protocols consistent with established guidelines [8,29].

Implementing a structured scheduling system for NORA has brought noticeable improvements in procedural coordination, resource utilization, and patient flow efficiency. Despite an observed 4% overall decline in total NORA cases, our findings align with global trends that highlight NORA’s expanding role in modern healthcare. Moreover, the scheduling model adopted in our institution serves as a practical application of evidence-based recommendations, emphasizing data-driven planning, staff training, and institutional alignment to enhance NORA service delivery. By supporting a feedback-based, scalable framework, this approach fosters continuous quality improvement, aligning with current literature on sustainable healthcare strategies [4,44].

## Conclusions

This study demonstrates how a structured NORA scheduling model can improve procedural coordination and service distribution in a tertiary hospital. Variations in departmental case trends - such as increases in angiography and nuclear medicine, and declines in GI and MRI - reflect changing clinical demands and improved procedural stratification. These findings underscore the importance of adaptive scheduling and resource planning in NORA services. Future work should include outcome-based analyses and predictive tools to further optimize NORA delivery and ensure continued safety and efficiency.
